# Comparative retrospective study of triangular osteosynthesis with and without robotic assistance for unilateral unstable sacral fractures combined with lumbosacral junction injuries

**DOI:** 10.1186/s12893-022-01857-9

**Published:** 2022-12-16

**Authors:** Zhao-Jie Liu, Ya Gu, Jian Jia

**Affiliations:** Department of Orthopaedics, Tianjin Hospital, 406 Jiefangnan Road, Tianjin, 300211 China

**Keywords:** Triangular osteosynthesis, Minimally invasive surgery, Robot, Sacrum, Fracture fixation, internal

## Abstract

**Background:**

To compare the clinical efficacy of unilateral unstable sacral fractures (USFs) involving the lumbosacral region treated with and without robot-aided triangular osteosynthesis (TOS).

**Methods:**

Patients of the unilateral USF combined with the ipsilateral lumbosacral junction injury (LSJI) treated with TOS were retrospectively analyzed and divided into two groups: the robot group (TOS with robotic assistance) and the conventional group (TOS with open procedure). Screw placement was assessed using the modified Gras criterion. Patients were followed up with routine visits for clinical and radiographic examinations. At the final follow-up, clinical outcomes were recorded and scored using the Majeed scoring system.

**Results:**

Eleven patients in the robot group and seventeen patients in the conventional group were recruited into this study. Significant differences in surgical bleeding (P < 0.001) and fluoroscopy time (P = 0.002) were noted between the two groups. Operation time (P = 0.027) and fracture healing time (P = 0.041) was shorter in the robot group. There was no difference in postoperative residual displacement between the two groups (P = 0.971). According to the modified Gras criterion, the percentages of grade I for sacroiliac screws in the two groups were 90.9% (10/11) and 70.6% (12/17), and for pedicle screws were 100% (11/11) and 100% (17/17), respectively. The rate of incision-related complications was 0% (0/11) in the robot group and 11.8% (2/17) in the conventional group. Statistical differences were shown on the Majeed criterion (P = 0.039), with higher scores in the robot group.

**Conclusion:**

TOS with robotic assistance for the treatment of unilateral USFs combined with ipsilateral LSJIs is safe and feasible, with the advantages of less radiation exposure and fewer incision-related complications.

## Background

Unstable sacral fractures (USFs) have a complete disruption of the posterior ring, most commonly in patients following high-energy trauma, including falls from height and traffic accidents. In this fracture type, the ipsilateral L5/S1 articular process can be involved due to the upward extension of a high transverse or longitudinal sacrum fracture 1. According to the description of lumbosacral junction injuries (LSJIs) initially from Isler [2], classification was summarized as follows: type I (extraarticular lesions), type II (articular lesions) and type III (complex lesions). Of type II lesions, three subtypes were classified with IIa (fractures of the S1 facet), IIb (fractures medial to the S1 facet) and IIc (with rotation of the hemipelvis and subluxation of the S1 facet).

Significantly displaced USFs, particularly with lumbosacral region involvement, predictably lead to spinopelvic instabilities with potentially adverse functional consequences, such as lumbosacral pain and gait changes. The purposes of treatment are to reconstruct the sacral anatomy and achieve the sufficient stability at the spinopelvic area for early mobilization. In recent years, sacroiliac screw or plate fixation, aimed at connecting and fixing the ilium and sacrum, is a reliable modality for posterior pelvic ring injuries with non-displacement, even for sacral fractures combined with type I LSJIs [3, 4]. However, if a USF is associated with a severer LSJI, sacroiliac screw or posterior plate fixation is inappropriate due to inability to stabilize the lumbosacral region.

Unilateral spinopelvic fixation for a vertically USF, called as distraction spondylodesis, was first described by Kach and Trentz [5]. As a modification, triangle osteosynthesis (TOS) has the devices by connecting the lumbar spine to the ilium and a supplemental sacroiliac screw, with the dual function of reducing and fixing sacral fractures. Compared with fixation of the sacroiliac screw and the posterior plate, TOS has significant advantages on biomechanics for the treatment of vertically USFs [6–9]. This construct can also indirectly stabilize the affected lumbosacral region by crossing the spinopelvic area.

However, literatures reveal that complication rates associated with their implantation are not insignificant. Owing to open reduction and soft tissue dissection, the reported deep infection rate was 8% and wound healing disturbance rate was as high as 26%, particularly in those patients with multi-planar sacral fractures requiring bilateral fixation [10–12]. In recent years, to overcome the shortcomings of the conventional open surgery, minimally invasive orthopedic surgery aimed to bring benefits, such as less bleeding and earlier postoperative rehabilitation. It’s still problematic to ensure that each screw is inserted accurately into the optimal area with the fluoroscopic free-hand technique, although percutaneous lumbopelvic fixation has been proved to be an effective method for stabilizing USFs. The incidence of mal-positioned sacroiliac screws has been reported to range from 2 to 15% [13, 14] and neurological injury from 0.5 to 7.7% [13, 15], making minimally invasive screw placement still a deliberate decision. In addition, both the patient and the surgeon are subject to large amounts of harmful radiation because the relationship between the guide pin and the bony landmarks in the surgical area needs to be clearly identified.

## Application of the orthopedic surgery robot

Consequently, the urgent requirement to reduce complications, avoid excessive radiation and improve screw placement accuracy caters to the development of computer navigation and robot-aided surgery [16, 17]. Although some studies on robot-aided pelvic fracture fixation have already been reported, minimally invasive TOS with robotic assistance for the treatment of USFs is still lacking. To date, TOS with and without robotic assistance has been performed in our constitution for patients with USFs involving ipsilateral lumbosacral region. The purposes of this study were to retrospectively analyze the data from these patients, compare the clinical efficacy, and discuss existing issues in the use of orthopedic surgery robots.

## Methods

### Patients

Inclusion criteria for this study were skeletally mature patients of unilateral USFs combined with ipsilateral LSJIs treated with robot-aided minimally invasive TOS or conventional open reduction and TOS fixation. Exclusion criteria were as follows: (1) the time from injury to surgery was more than 4 weeks; (2) patients whose S1 vestibula is too narrow to accommodate a cannulated screw with the diameter of 6.5 mm, (3) the injured sacral nerves need to be decompressed, (4) the follow-up period was less than 12 months.

Prior to the application of the orthopedic surgery robot at our institution, for patients who met the inclusion criteria, TOS was performed using the traditional open procedures. Thereafter TOS with robotic assistance has been performing in patients of unilateral unstable sacral fractures combined with lumbosacral junction injuries. All patients were divided into two groups: the robot group (minimally invasive TOS with robotic assistance) and the conventional group (open reduction and TOS fixation).

Skeletal tractions were performed in patients with vertically USFs to reduce and resist cephalad migration of the hemipelvis. X-ray films and three-dimensional (3D) computed tomography (CT) scans were routinely obtained. Vestibular anatomy of S1 was measured with CT scans to ensure if a cannulated screw with the diameter of 6.5 mm can pass through. Surgical treatment was performed once the patient was stable both physiologically and hemodynamically.

### Surgical equipment

**Fig. 1:**
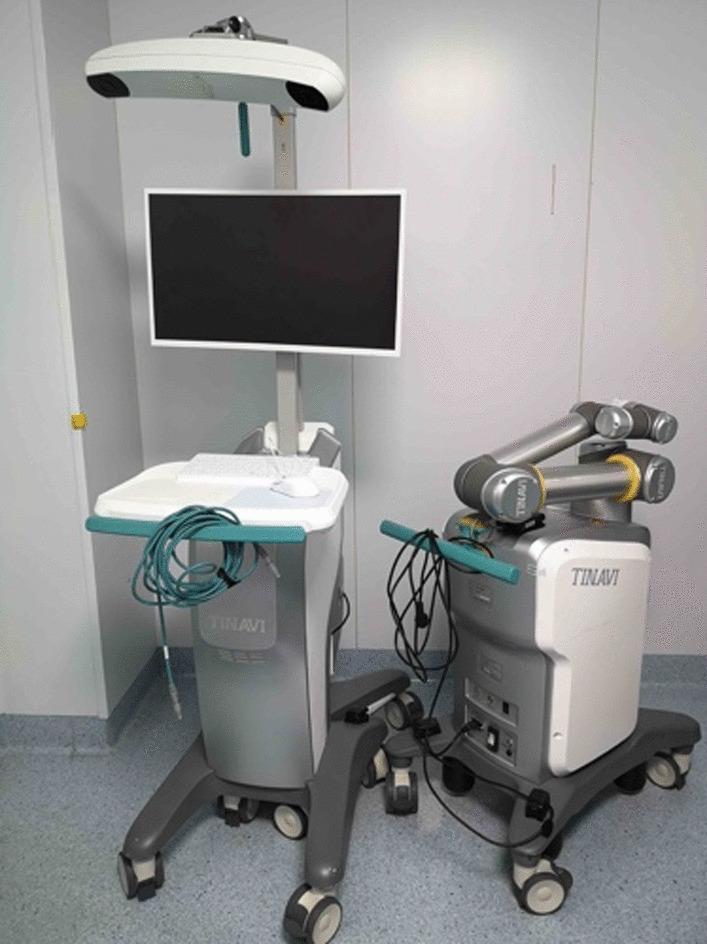
The TiRobot system

The TiRobot system (TINAVI Medical Technologies, Beijing, China) for orthopaedic surgery was made in China, containing a main console with a surgical planning and controlling workstation, an optical tracking system, and a robotic arm with six joints (Fig. 1). The C-arm machine was produced by Siemens (Germany). The pedicle screws, iliac screws, rods and cannulated screws were produced by Kanghui (China).

### Surgical methods

#### The surgical procedure of patients in the robot group

The patient was in the prone position on a radiolucent operation table after general anesthesia. We established a sterile working environment for the robotic arm and assembled a locator at the end of the robotic six-joint-arm.

**Fig. 2:**
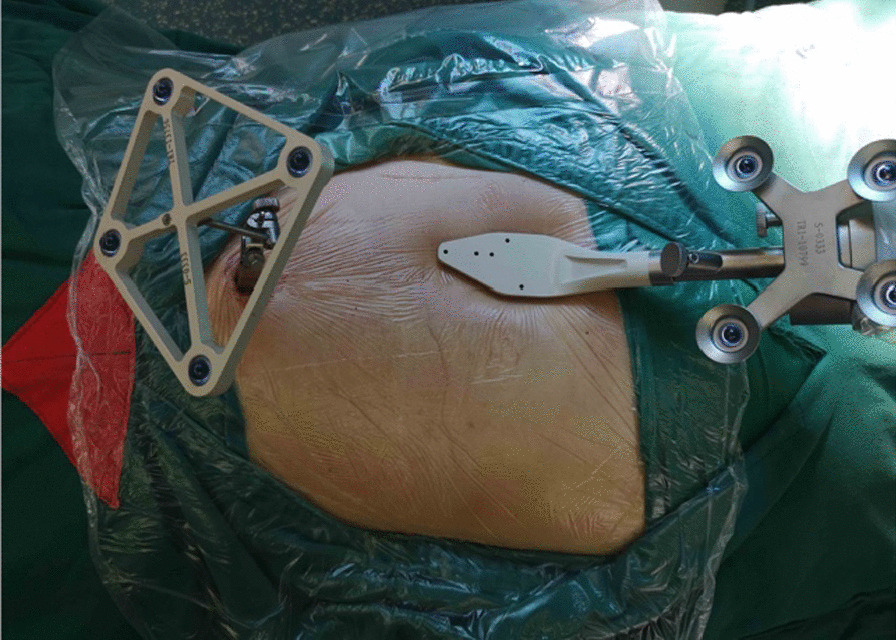
The positioning device (the locator) on right and the tracker on left

**Fig. 3:**
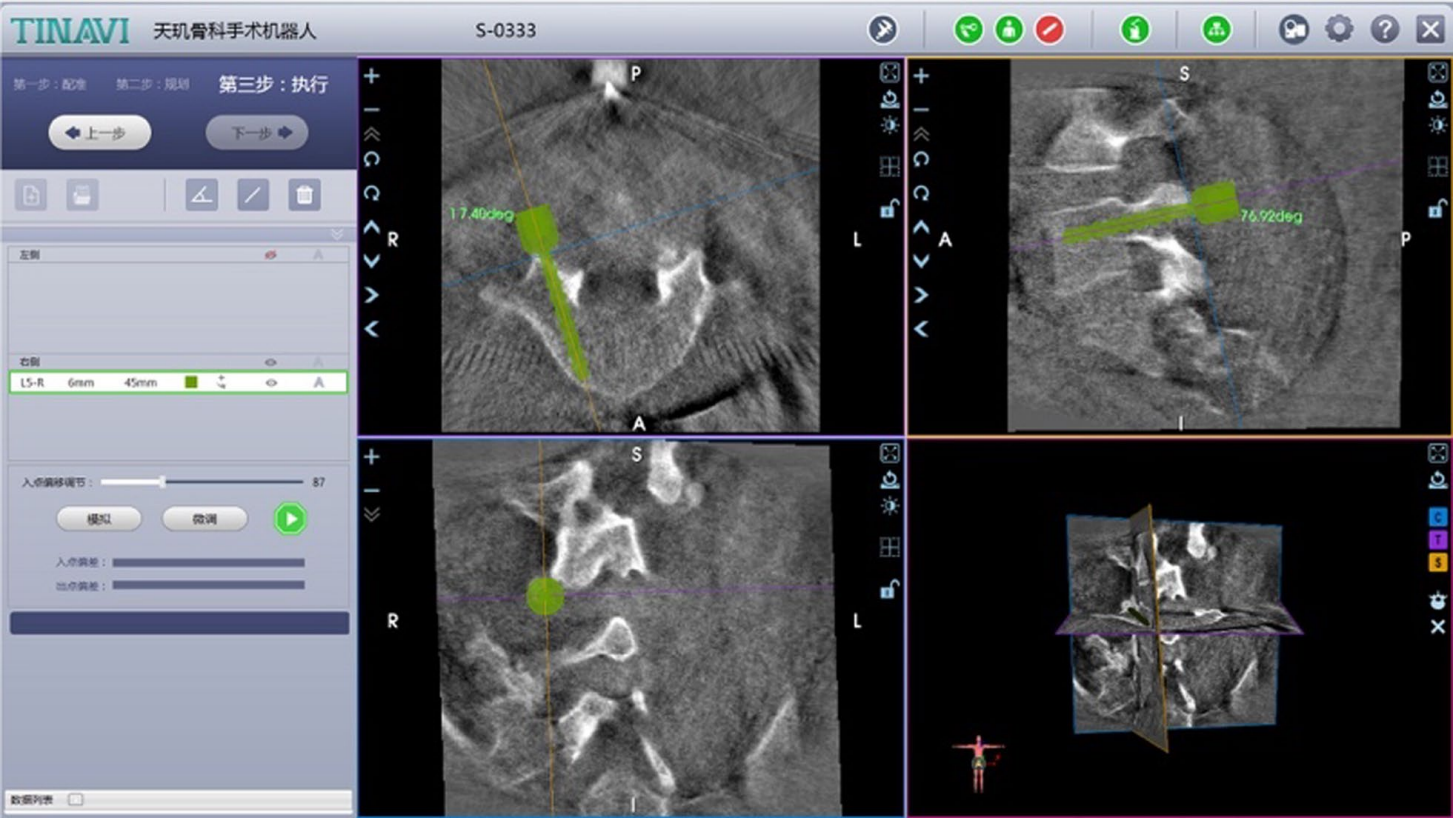
Trajectory planning of the unilateral pedicle screw on L5

**Fig. 4:**
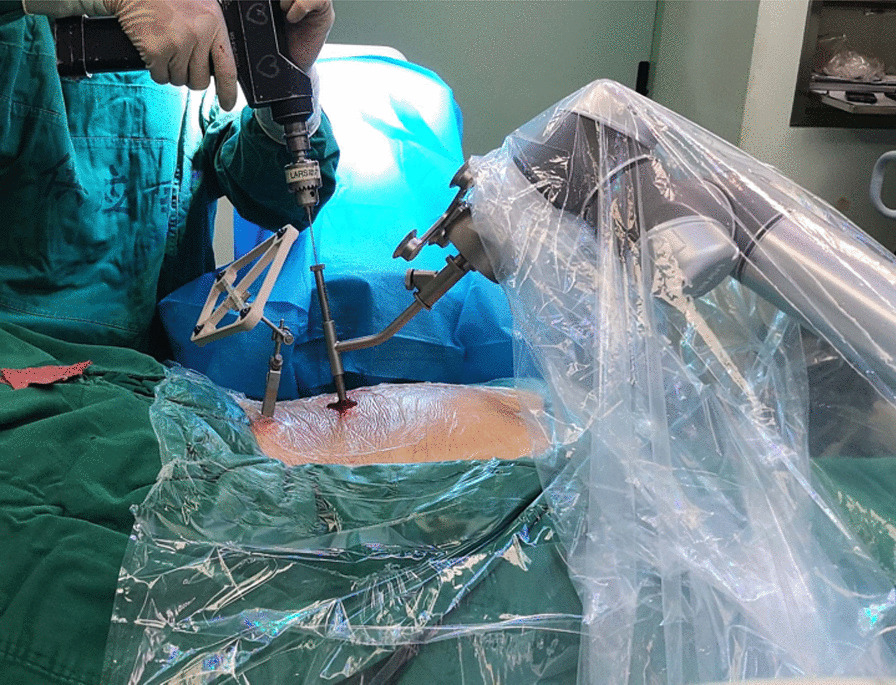
A guide pin is being drilled under guidance of the robotic arm

**Fig. 5:**
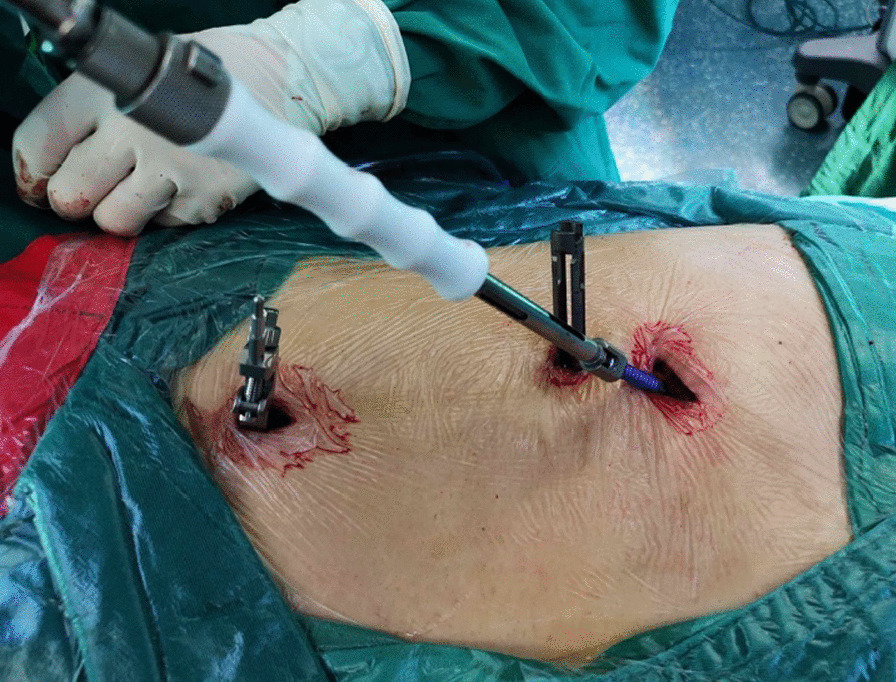
Implantation of a polyaxial iliac screw from the entry point of PSIS

**Fig. 6:**
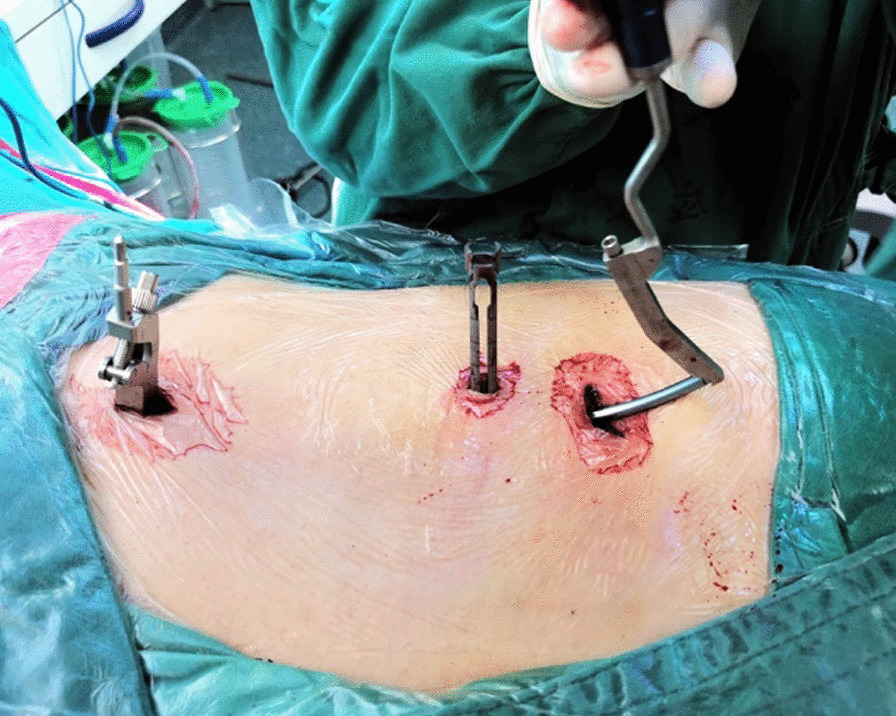
Percutaneous insertion of a connection rod

**Fig. 7:**
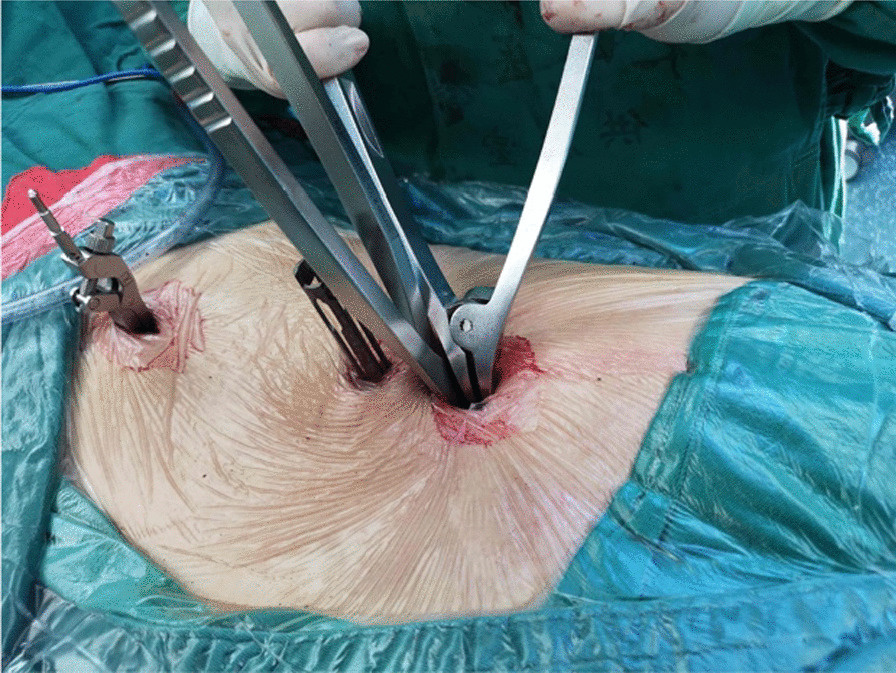
Closed reduction of the sacral fracture

First, we fixed a spinal tracker on L3 spinous process through a 2-cm incision and moved the locator onto the skin at the surgical area (Fig. 2). After collecting the intraoperative fluoroscopy with the 3D C-arm machine, we transmitted the data into the TiRobot workstation. Next, the trajectory of L5 pedicle screw was planned at the workstation (Fig. 3). We removed the locator and assembled a guider with a pilot sleeve, and then pressed the start button on the main console. The arm will move to the surgical area according to the command, and automatically complete the screw positioning. Before insertion of the guide pin, it is necessary to ensure that the pilot sleeve can smoothly contact the bone through a 1-cm skin incision. After confirming the trajectory accuracy on the workstation, a guide pin was drilled into the L5 pedicle (Fig. 4). In order to keep the consistency of the actual and planned trajectory, TiRobot was able to recalibrate the trajectory repeatedly in the process of the guide pin insertion, maintaining the positioning accuracy less than 1-mm. After verifying the pin’s position with fluoroscopic images, we fixed a polyaxial cannulated pedicle screw with a diameter of 6-mm and the length of 4.5- to 5-cm. The following step was to expose the ipsilateral posterior superior iliac spine (PSIP) through another 3-cm incision. After resecting part of the bone to avoid skin irritation caused by the screw, we implanted a polyaxial iliac screw with a diameter of 7-mm and the length of 8- to 10-cm along the medial and lateral lamina, keeping the direction to the anterior inferior iliac spine (Fig. 5). A contoured rod was percutaneously inserted to engaged the L5 pedicle screw and the iliac screw (Fig. 6). If there is rotational displacement of the pelvic ring, a partial threaded Schantz pin in the iliac crest, as a "joy stick", can be temporarily used for reduction. For sacral fractures with vertical displacement, it is critical to apply longitudinal traction and the distraction clamp (Fig. 7). Once reduction was complete, all connectors were locked.

**Fig. 8:**
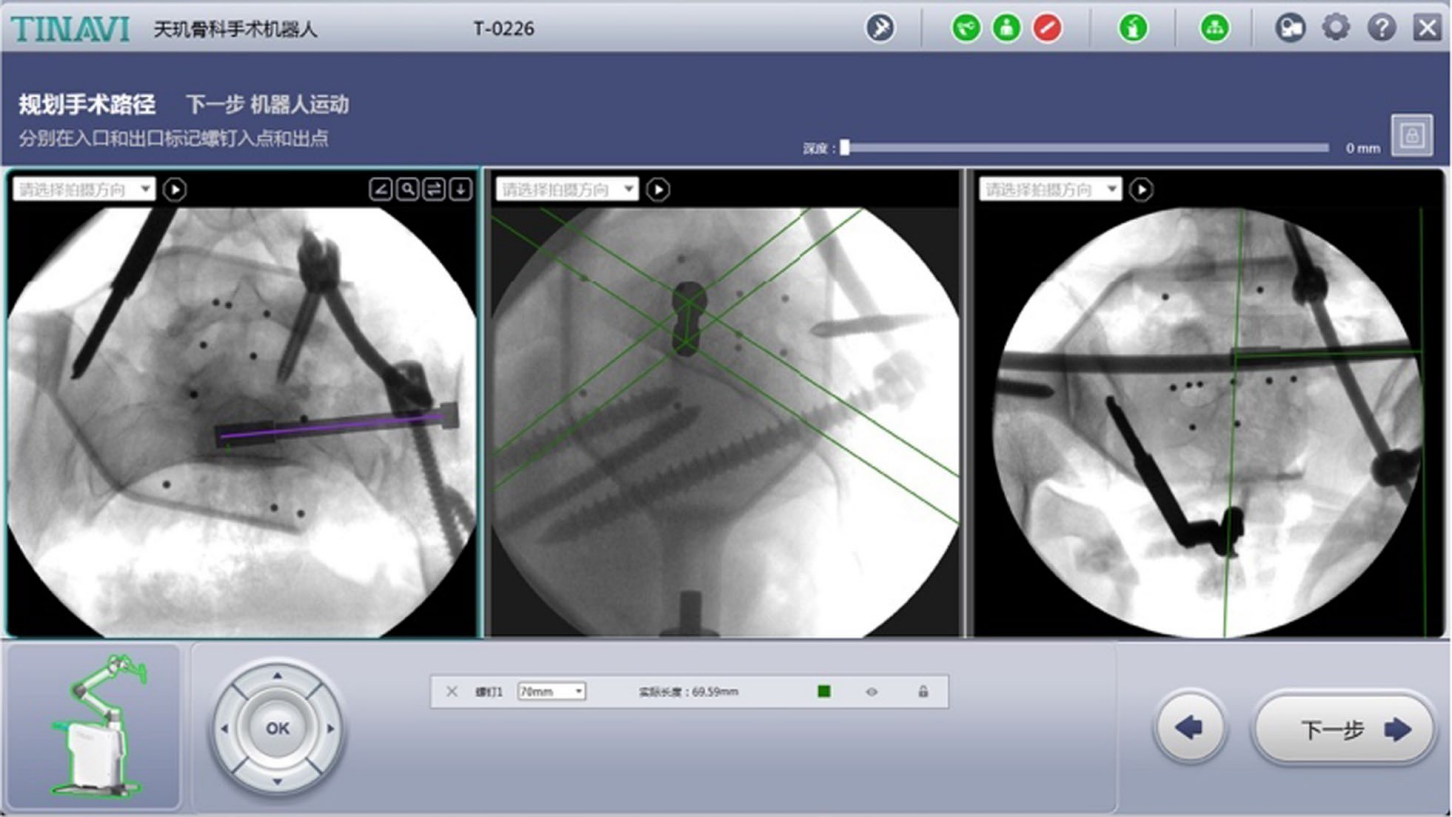
Trajectory planning of the ipsilateral sacroiliac screw on S1

**Fig. 9:**
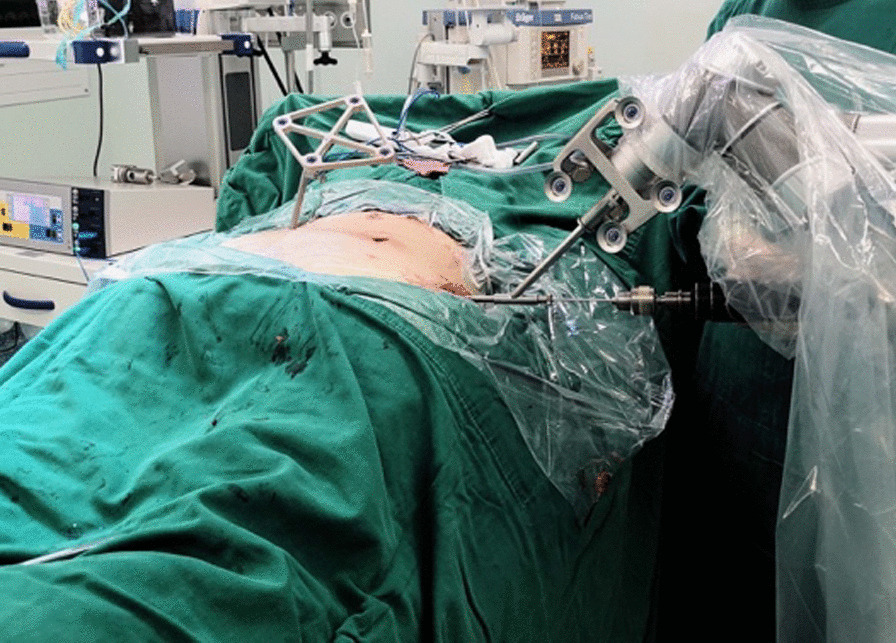
Insertion of a guide pin along the S1 screw corridor with TiRobot assistance

In the process of planning the sacroiliac screw, we assembled a pelvic locator at the end of the robotic arm and then fixed a matching tracker percutaneously at the contralateral PSIP. Intraoperative pelvic images, including inlet, outlet and lateral views, were obtained using the C-arm machine and imported into the workstation for planning the trajectory of the S1 screw (Fig. 8). After that, we drilled a guide pin into the S1 corridor with robotic assistance (Fig. 9). A cannulated screw with an appropriate length was then implanted to eliminate the fracture gap and maintain the horizontal stability. It is critical to note that all connectors need to be loosened and retightened in the end for relieving the longitudinal distraction acting on the L5/S1 disk. Finally, after the satisfactory position of the fractures and the implants was confirmed using the C-arm machine, all the incisions were irrigated and sutured. If the affected L5/S1 joint cannot be anatomically reconstructed, fusion at the lumbosacral region is required by bone graft.

#### The surgical procedure of patients in the conventional group

Patients in the conventional group were placed in the same prone position. The L5/S1 facet joint, the ipsilateral posterior superior iliac spine (PSIS), and the partial sacral fracture were exposed through a posterior median longitudinal incision with the length of 10- to 12-cm. The L5 pedicle screws and the iliac screws were inserted under direct vision, and then reduction was performed. Finally, under fluoroscopy of the C-arm machine, a guide pin was drilled with the free-hand technique, followed by implantation of an appropriate cannulated sacroiliac screw through a 1-cm incision. In addition, fusion of the L5/S1 joint on the affected side is to be performed according to the same principles as in the robot group.

### Postoperative management

Both groups of patients underwent the identical treatment. Within 48 h after surgery, the first-generation cephalosporin was routinely administered to prevent wound infection. Immediate activities, such as sitting up, were encouraged. Then all patients were instructed on the muscle strength and range of motion of the joint. In addition to special patients with associated concomitant injuries, partial weight-bearing with a walking aid was allowed in patients 2–4 weeks postoperatively. The intraoperative and postoperative critical indictors were recorded. All patients were followed up once a month until the bone union and every three months thereafter. X-ray films were obtained at each follow-up.

Maximum residual displacement was evaluated according to the postoperative radiograph examination and was classified as excellent (0–5 mm), good, (6–10 mm), fair (11–15 mm), and poor (> 15 mm) by Lindahl et al. [18]. The position of the pedicle and sacroiliac screw was assessed with postoperative CT scans based on the modified Gras criterion [19], which includes as follows: grade I, a screw is totally in the cancellous bone (satisfactory position); grade II, a screw is still in the bone structures, cutting the cortical bone (secure position); grade III, a screw has penetrated the cortical bone (misplaced position). Fracture healing was evaluated based on the radiological findings at each follow-up visit. Bone healing can be confirmed if there is continuous callus across the fracture end. In addition, blurring of the fracture line or disappearance of the fracture gap on imaging is also a criterion for determining healing. The clinical outcomes were investigated with Majeed’s scoring system (excellent (85–100), good (70–84), fair (55–69), and poor (< 55)) [20] at the final follow-up at least 12 months after surgery.

### Statistical analysis

Statistical analysis was performed using SPSS version 20.0 (SPSS Inc., Chicago, IL, USA). Measurement data such as age were presented as mean ± standard deviation (SD) and compared using independent samples t-test. Count data such as gender were compared using Chi-square test or Fisher analysis. A statistically significant difference was accepted for P-values < 0.05.

## Results

Referring to inclusion and exclusion criteria, a total of 28 patients were retrospectively analyzed from March 2010 to January 2021. There were 11 patients in the robot group and 17 patients in the conventional group. The most common mechanisms of injury were falls from heights (17 cases) and vehicle accidents (8 cases), and 3 cases were caused by crush injuries. All patients combined anterior pelvic ring injuries, including pubic rami fractures in 22 patients, a simple symphyseal disruption in 4 patients, and a symphyseal disruption with rami fractures in 2 patients. Five patients had neurologic impairment to some component of the sacral plexus, with grade II of Gibbons classification [21]. According to Tile classification of pelvic fractures [22], there were 5 patients with type B and 19 patients with type C. The demographic data of the two groups revealed no significant difference in this study (Table 1).

**Table 1:** Patients’ demographics and characteristics.

Characteristics	Robot group (n=11)	Conventional group (n=17)	*t/X*^*2*^	*P* value
Age(year), M±SD	40.7±12.7	39.8±11.2	*t*=0.211	0.834
Gender, n(%)			*X*^*2*^=0.312	0.705
Male	7(63.6%)	9(52.9%)		
Female	4(36.4%)	8(47.1%)		
Isler classification, n(%)			*X*^*2*^=1.315	0.613
I	1(9.1%)	3(17.6%)		
II	7(63.6%)	12(70.6%)		
III	3(27.3%)	2(11.8%)		
Tile classification, n(%)			*X*^*2*^=0.368	0.653
Type B	3(27.2%)	3(17.6%)		
Type C	8(72.8%)	14(82.4%)		
Time to surgery(day)	7.3±5.6	7.2±4.7	*t*=0.049	0.961
Follow up (month)	18.9±5.3	18.1±6.1	*t*=0.348	0.730

In both groups, all USFs involving the lumbosacral regions were reduced and stabilized with TOS. Since anatomical reconstruction of the injured L5/S1 facet joint was not possible in 3 patients in the robot group and 6 patients in the conventional group, fusion was performed with the autogenous bone graft from the ipsilateral PSIS. Of 22 patients with ramus fractures, 11 were treated with open reduction and plating fixation through the Stoppa approach while the rest underwent closed reduction percutaneous superior pubic ramus screw or anterior subcutaneous internal fixator (INFIX) fixation. The symphyseal disruption in 4 patients was stabilized with the construction locking plate via the Pfannenstiel approach. The two patients of symphyseal disruption with associated rami fractures underwent open reduction and plating fixation through the Stoppa approach combined with the lateral window of the ilioinguinal approach.

No intraoperative neurovascular damage occurred in all patients. The intraoperative and postoperative indicators of the two groups were compared (Table 2). The average operation time of posterior pelvic ring (disinfection to closure) was 94.6 ± 21.8 min in the robot group and 112.7 ± 18.6 min in the conventional group, respectively (P = 0.027). The amount of surgical bleeding was 98.2 ± 36.1 ml in the robot group and 286.8 ± 92.7 ml in the conventional group, revealing a significant difference in this study (P < 0.001). The intraoperative fluoroscopy times were 24.1 ± 6.3 in the robot group and 34.1 ± 8.4 in the conventional group with a significant difference (P = 0.002). Patients in the conventional group had a longer hospital stay, compared to the robot group (P = 0.044).

**Table 2:** Results comparison between the two groups

Characteristics	Robot group	Conventional group	*t/X*^*2*^	*P* value
Operation time of the posterior pelvic ring (min), M±SD	94.6±21.8	112.7±18.6	*t*=-2.347	0.027
The amount of surgical bleeding (mm)	98.2±36.1	286.8±92.7	*t*=-6.408	<0.001
Intraoperative fluoroscopy times, M±SD	24.1±6.3	34.1±8.4	*t*=-3.386	0.002
Maximum residual displacement			*X*^*2*^=1.056	0.543
Excellent (0-5 mm)	9(81.8%)	16(82.4%)		
Good (6-10 mm)	2(18.2%)	1(17.6%)		
Gras criterion on evaluation of sacroiliac screw position, n(%)			*X*^*2*^=1.638	0.355
Grade I	10(90.9%)	12(70.6%)		
Grade II and III	1(9.1%)	5(29.4%)		
Hospital stay(day)	14.2±5.4	21.0±8.4	*t*=-2.133	0.044
Healing time of sacral fractures (month)	3.7±1.5	4.9±1.2	*t*=-2.173	0.041
Majeed score	90.7±5.6	85.5±5.6	*t*=2.199	0.039

Postoperative maximum residual displacement of the pelvic fractures in the robot group was excellent in 9 patients and good in 2 patients, while that in the conventional group was 14 and 3, respectively (P = 0.971). According to the screw position evaluated with the modified Gras criterion, implanted pedicle screws in all patients were in the cancellous bone, meeting grade I (satisfactory position). There were 10 sacroiliac screws with grade I and 1 with grade II in the robot group, and 12 with grade I and 4 with grade II (secure position) in the conventional group. One sacroiliac screw in the conventional group penetrated the cortical bone (grade III, misplaced position), without clinical symptoms of vascular or neurovascular impairment. All the L5 pedicle screws were in the cancellous bone with grade I in the two groups. No statistical difference was shown on the screw position between the two groups (P = 0.355), but the rate of grade I was 90.9% (10/11) vs 70.6% (12/17).

Three patients (2 in the robot group and 1 in the conventional group) complained of slight pain at the region of PSIS in the supine position due to prominent implants. The rate of the complication relating to the incision was 0% (0/11) in the robot group and 11.8% (2/17) in the conventional group. No wound infection was noted in the robot group, while one patient in the conventional group suffered with a deep infection. After thorough debridement and frequent dressing changing, purulence was eliminated. In the conventional group, local hematoma in the deep wound developed in one patient, and the wound healed in two weeks after puncture and drainage.

No fracture reduction loss and hardware loosening were observed during follow-ups. After 3–6 months’ conservative treatment with oral medication, 4 out of 5 patients with initial neurologic impairment achieved full recovery, while the remaining one patient revealed partial improvement at the final follow-up. All sacral fractures appeared as bone union within 6 months. The mean healing time was 3.7 ± 1.5 months in the robot group and 4.9 ± 1.2 months in the conventional group, with the healing time exhibiting a statistical difference (P = 0.041). The Majeed functional score at the final follow-up was 90.7 ± 5.6 in the robot group and 85.5 ± 5.6 in the conventional group (P = 0.039). Unable to tolerate the heavy physical work as usual, one patient in the robot group and two patients in the conventional group had to change jobs, mainly because of postoperative pain in the lumbosacral region.

**Fig. 10:**
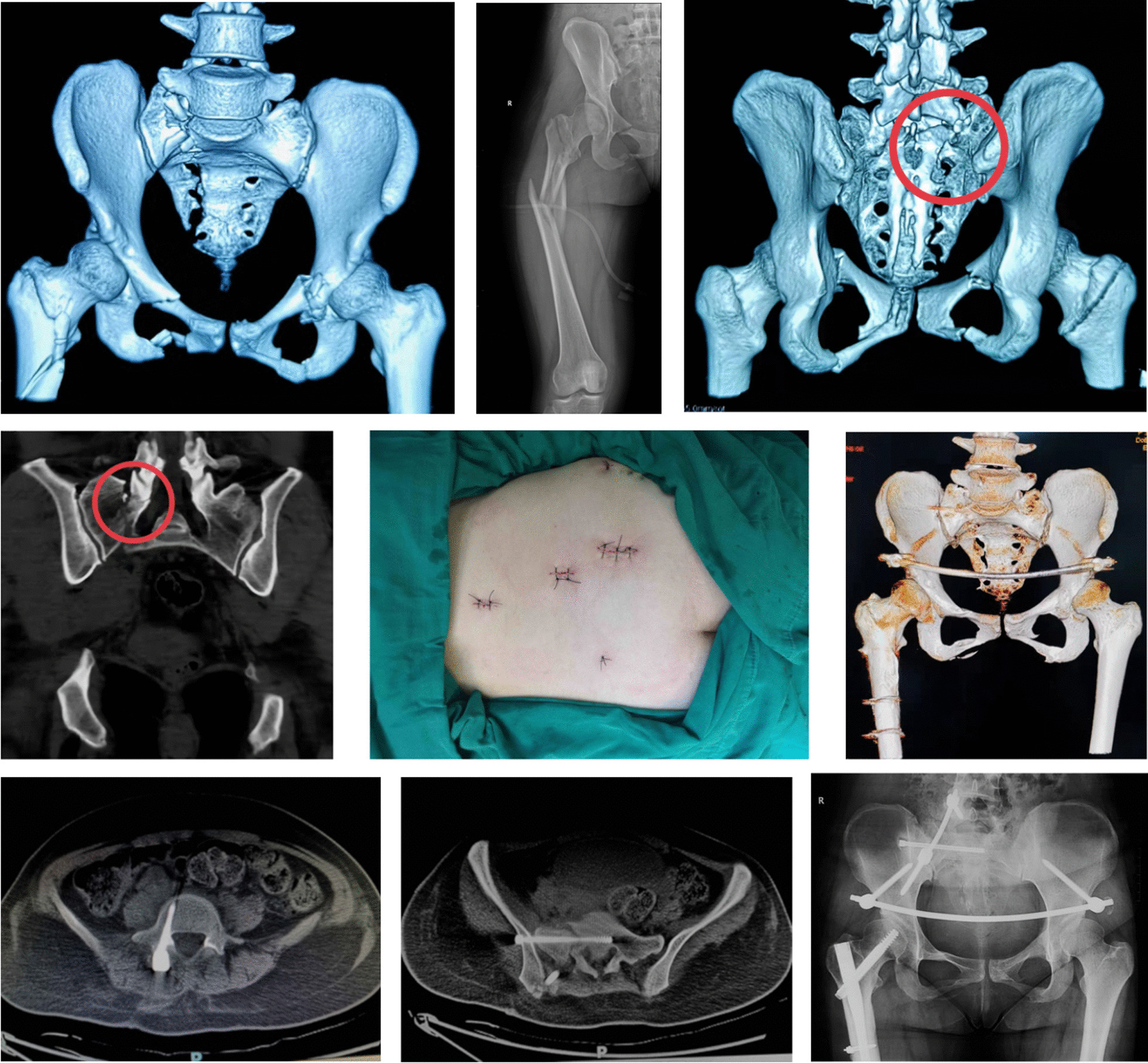
Female, 34-year-old, a fall from height. A The CT reconstruction view on admission showed a vertically displaced fracture on right sacrum associated with bilateral rami fractures (Tile type C1). B The radiograph showed a comminuted subtrochanteric fracture on right femur. C,D The areas in the red circles on CT views revealed a fracture on right S1 articular process (Isler type IIb). E The surgical incisions of posterior pelvic ring after minimally invasive TOS with TiRobot assistance. F Postoperative CT reconstruction image. The anterior pelvic ring was stabilized by INFIX and the satisfactory reduction of the pelvic fracture and the femoral fracture was achieved. G,H Axial CT view demonstrated the pedicle screw and the sacroiliac screw were both in the cancellous bone. I Postoperative 6-month radiograph showed fracture healing

**Fig. 11:**
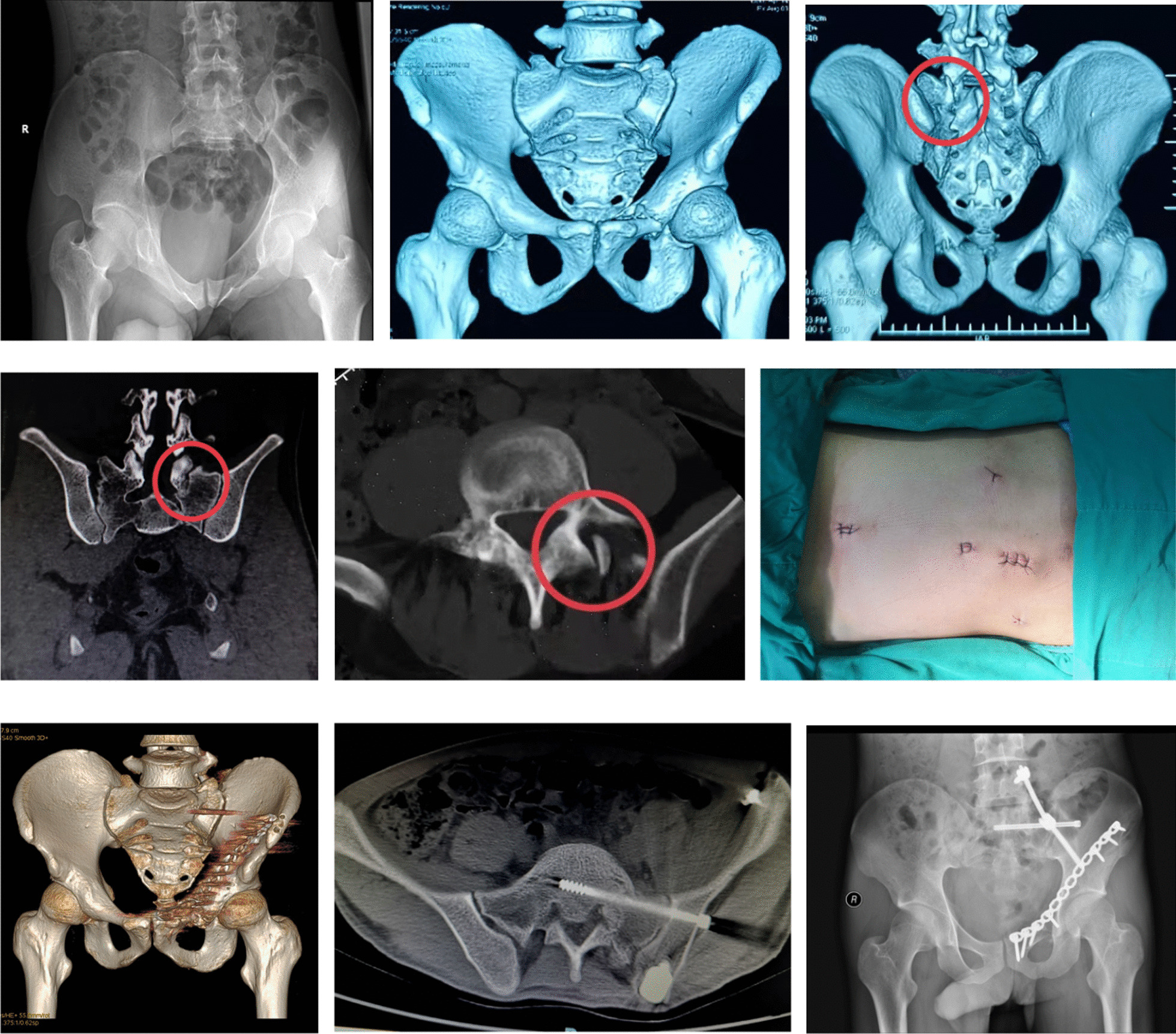
Male, 26-year-old, a crush injury. A. Radiograph on admission showed a displaced pelvic fracture due to the lateral compression mechanism. B The CT reconstruction image revealed an internally rotated displaced fracture on left sacrum combined with ipsilateral superior ramus fracture (Tile type B2). C-E The areas in the red circles on CT views revealed the fracture and subluxation at the level of L5/S1 facet joint (Isler type IIc). F The skin incisions of posterior pelvic ring after minimally invasive TOS with TiRobot assistance. G CT reconstruction taken postoperatively. H Postoperative axial CT view of S1 demonstrated a satisfactory position of the sacroiliac screw. I Postoperative 6-month radiograph showed fractures healing

Typical cases are shown in Figs. 10 and 11.

## Discussion

In this study, we reviewed data from patients with unilateral USF involving the ipsilateral lumbosacral region and compared the treatment outcomes of these patients underwent TOS with or without robotic assistance.

The lumbosacral junction is a special region that transfers upper body weight to the pelvis and lower extremities through the sacrum. The conduction of axial stress can be seriously affected due to the damage of the L5/S1 joint. There is a general consensus among orthopedic surgeons worldwide regarding TOS in the treatment of vertically USFs, especially with lumbosacral region involvement [5–10]. To avoid excessive dissection of soft tissue, controllability and precision of minimally invasive surgery on pelvic fractures with the free-hand technique remains a major challenge, despite considerable progress. Until recently, pelvic fracture surgeries with robotic assistance demonstrate significant benefits for patients, with some studies revealing that robot-aided surgeries had higher accuracy for screw implantation and lower radiation exposure than fluoroscopy-guided free-hand surgeries [23]. In a study of 33 osteoporosis patients with sacral fractures, the authors compared robot-aided to conventional free-hand sacroiliac screw fixation. They found that robotic assistance led to higher rates of screw placement accuracy, with the robot-aided group demonstrating an accuracy of 94.4% (vs 73.3%), using the modified evaluation criterion set by Gras et al. [17].

In our study, the actual positions of the pedicle and sacroiliac screws in both groups were compared with the planned trajectories to assess their accuracy. Based on the evaluation of postoperative CT images, we found that all the pedicle screws were within the vertebral bodies, indicating that precise placement of the L5 pedicle screws was relatively easy even without the assistance of the TiRobot. There were 5 sacroiliac screws cutting the cortical bone and one sacroiliac screw penetrating the cortex in the conventional group, while all the screws in the robot group were in the cancellous bone. Due to the limited cases in this study, there was no statistical difference in the accuracy of screw implantation. However, the percentage of Gras grade I in the two groups was still significantly discrepant (90.9% vs 70.6%), indicating that insertion of the sacroiliac screw can be very demanding with the free-hand technique. In the process of guide pin implantation, it’s tough to slightly adjust the pin’s direction, and frequent drillings will increase the risk of the neurovascular injury and have a direct impact on the fixation strength. Consequently, most surgeons are unwilling to adjust the guide pin when using the free-hand technique once the position is barely acceptable. Even so, the mean operation time in the robot group is much shorter than that in the conventional group (P = 0.027).

In addition, patients and medical staff are exposed to large amounts of radiation in the operating room, as repeated fluoroscopy is often required to determine the safe trajectory of the guide pin when it is being drilled. According to the recorded frequency of intraoperative fluoroscopy in this study, the data were significantly lower in the robot group (P < 0.001), which was similar to the existing reports using the same technique [16, 17, 24]. The majority of guide pins in the robot group were successfully implanted at one time, while that in the conventional group need to be adjusted twice or more.

Minimally invasive surgery is normally performed with indirect reduction to avoid excessive dissection of soft tissue, reducing the amount of bleeding and shortening the suture time. In this study, the mean length of the surgical incision in the robot group was much smaller than that in the conventional group, demonstrating the advantages of the robot-aided surgery. For each patient in the conventional group, we made a posterior median longitudinal incision with the length of 10–12 cm followed by the dissected paravertebral muscles for screw implantation under direct vision, which inevitably led to more dissection than that in the robot group. In addition, further destruction of blood supply at fracture sites was avoided by using closed reduction, which may be the main reason why the fracture healing time in the robot group was shorter than that in the conventional group (P < 0.041). Although no significant difference was noted, the complication rate relating to the incision in the robot group was much lower than that in the conventional group (0% vs 11.8%). In our view, a few cases may have an impact on the analysis of clinical effects, but minimally invasive surgery is, after all, much less invasive than conventional open surgery.

Gardner et al. reported that the S1 screw corridor is on average of 36% narrower in the dysmorphic sacra compared with the normal [25]. The presence of a dysmorphic sacrum is a risk factor for inaccurate placement of sacroiliac screw in S1. Accordingly, preoperative clarification of the sacral morphology, particularly the dimensions of the sacral vestibule, is essential to determine whether a cannulated screw with the diameter of 6.5 mm can be accommodated, even though robot-assisted screw placement is more accurate than that with the free-hand technique [16, 17]. Different from the traditional navigation system, the TiRobot system can monitor the trajectory in the process of guide pin implantation and identify the errors in real time. Also, it only takes approximately one minute to re-plan the screw path in case of the guide pin with an unsatisfactory position. Different from the matching failure in CT or 3D fluoroscopic navigation, screw planning with the TiRobot system will not be affected by fracture displacement, as long as the tracker on the patient side can consistently identify by the optical tracking system [26].

As an upgrade to the navigation, TiRobot is not omnipotent. After all, it doesn’t have the reduction function. Poor reduction can significantly reduce the 3D available space of the screw corridor. Improving the reduction quality, especially the anatomical reduction at the narrowest part of the corridor, is an important guarantee for safe and effective screw placement. Also, robot-aided orthopedic surgery is not without its disadvantages. TiRobot is a bit of cumbersome with high initial costs, and manipulation necessitates the cooperation of professionals and additional trained medical personnel. The lack of reliable tactile feedback system can only be compensated by surgeons’ visual information, which is a common deficiency of current surgical robots, including TiRobot.

This study, however, has several limitations. Robot-aided surgery is still mostly confined to some large hospitals and has, to date, not seen wide adoption in orthopedics. In addition, the cases demonstrated are all in our institution, and too small sample size in this study may lead to the inaccuracy of outcome comparison. More comparative studies between robot-aided surgery and conventional open surgery were needed in multicenter for better evidence of USFs involving lumbosacral region. Therefore, further clinical assessment necessitates to improve the authority of these conclusions and define its future applications, limitations, and developed directions.

## Conclusion

Compared with the conventional open procedure, minimally invasive TOS with robotic assistance can be an effective treatment for the unilateral USF combined with the ipsilateral LSJI, with the significant advantages of less damage and less incision-related complications. When considering direct benefits, high accuracy and low radiation dependence, orthopedic robots are more recommended.

## Data Availability

The datasets used or analyzed during the current study are available from the corresponding author on reasonable request.

## Abbreviations

TOS: Triangular osteosynthesis
USFs: Unstable sacral fractures
LSJIs: Lumbosacral junction injuries
CT: Computed tomography
3D: Three dimensional
PSIS: Posterior superior iliac spine
INFIX: Anterior subcutaneous internal fixator
